# Robotic versus Open Partial Nephrectomy: A Systematic Review and Meta-Analysis

**DOI:** 10.1371/journal.pone.0094878

**Published:** 2014-04-16

**Authors:** Zhenjie Wu, Mingmin Li, Bing Liu, Chen Cai, Huamao Ye, Chen Lv, Qing Yang, Jing Sheng, Shangqing Song, Le Qu, Liang Xiao, Yinghao Sun, Linhui Wang

**Affiliations:** 1 Department of Urology, Changhai Hospital, Second Military Medical University, Shanghai, P. R. China; 2 Department of Radiology, Changhai Hospital, Second Military Medical University, Shanghai, P. R. China; 3 Department of Special Clinic, Changhai Hospital, Second Military Medical University, Shanghai, P. R. China; H. Lee Moffitt Cancer Center & Research Institute, United States of America

## Abstract

**Objectives:**

To critically review the currently available evidence of studies comparing robotic partial nephrectomy (RPN) and open partial nephrectomy (OPN).

**Materials and Methods:**

A comprehensive review of the literature from Pubmed, Web of Science and Scopus was performed in October 2013. All relevant studies comparing RPN with OPN were included for further screening. A cumulative meta-analysis of all comparative studies was performed and publication bias was assessed by a funnel plot.

**Results:**

Eight studies were included for the analysis, including a total of 3418 patients (757 patients in the robotic group and 2661 patients in the open group). Although RPN procedures had a longer operative time (weighted mean difference [WMD]: 40.89; 95% confidence interval [CI], 14.39–67.40; p = 0.002), patients in this group benefited from a lower perioperative complication rate (19.3% for RPN and 29.5% for OPN; odds ratio [OR]: 0.53; 95%CI, 0.42–0.67; p<0.00001), shorter hospital stay (WMD: −2.78; 95%CI, −3.36 to −1.92; p<0.00001), less estimated blood loss(WMD: −106.83; 95%CI, −176.4 to −37.27; p = 0.003). Transfusions, conversion to radical nephrectomy, ischemia time and estimated GFR change, margin status, and overall cost were comparable between the two techniques. The main limitation of the present meta-analysis is the non-randomization of all included studies.

**Conclusions:**

RPN appears to be an efficient alternative to OPN with the advantages of a lower rate of perioperative complications, shorter length of hospital stay and less blood loss. Nevertheless, high quality prospective randomized studies with longer follow-up period are needed to confirm these findings.

## Introduction

Nephron-sparing surgery (NSS) has been recommended as the reference standard of care for localized renal cell carcinoma due to equivalent oncological, improved functional outcomes and better long-term survival compared to those of radical nephrectomy [1]–[4]. With the advent of minimally invasive surgery, laparoscopic partial nephrectomy (LPN) has become a viable alternative to open surgery for small renal masses with the advantage of faster recovery. However, LPN is a kind of procedure with highly technical demanding and has a steep learning curve. A population based study by Abouassaly revealed that the introduction of laparoscopy in renal surgery has decreased uptake and use of partial nephrectomy for renal cell carcinoma at least partially due to technical ease and decreased surgical morbidity in laparoscopic radical nephrectomy [5].

Robotic-assisted laparoscopy allows for improved dexterity, high-definition, three-dimensional optics, tremor filtration, and an ergonomic setting to enhance surgeon comfort. A recently published systematic review and meta-analysis of RPN versus LPN shows that the RPN series offers a significantly less warm ischemic time than with an LPN procedure [6]. And numerous reports have demonstrated that RPN is feasible and safe for large (>4–7 cm) tumors, high complex or hilar tumors [7]–[9]. Therefore, the robotic surgical system duplicates the techniques of LPN and OPN. As the indications of RPN has been expanded and at some institutions RPN has replaced OPN as the preferred technique, more and more investigators focus their interests on the comparative outcomes of RPN versus OPN [10]–[13].

The aim of this study is to systematically search and analyze the currently available literature to compare the surgical outcomes of RPN with OPN.

## Methods

### Search strategy and study selection

The systematic review was done according to the Cochrane review guidelines. A systematic literature search was done using the electronic database including Pubmed, Web of Science and Scopus. Searches were performed in [Title/Abstract/Topic Subject] with the following terms of *“open”, “robotic/robot-assisted”, and “partial nephrectomy”*. Searches were restricted to publications in English. The electronic search was done in October 9, 2013.

Article selection was according to the Preferred Reporting Items for Systematic Reviews and Meta-analysis of Observational Studies in Epidemiology Recommendations for studies reporting. All relevant randomized controlled trials (quasi-randomized studies, such as those allocating by using alternate days of the admission date, were also included) and retrospective comparative studies (cohort or case-control studies) comparing robotic partial nephrectomy (RPN) with open partial nephrectomy (OPN) were included for further screening. Studies comparing OPN, LPN and OPN were also included as long as the data of RPN and OPN could be extracted. Review articles, case reports, conference paper, short survey, note editorials, letters to the editor, and animal experimental studies were excluded. When multiple reports describing the same population were published, the most recent or complete report was used. Two independent reviewers completed this process and all disagreements were resolved by their consensus.

### Data extraction and outcomes of interest

Studies comparing RPN with OPN were included. Articles comparing OPN, LPN and OPN were also included as long as the data of RPN and OPN could be extracted. Two independent authors reviewed the full texts of the included studies. Patient demographic characteristics, peri- and post- operative outcomes between the two procedures were compared. The following data was extracted from each eligible study: patients demographics, tumor size and nephrometry score, operative time, ischemia time, estimated blood loss, transfusion rate, conversion rate and complication rates. Postoperative complications were captured during the inpatient setting and within 30 days after surgery, and classified according to the Clavien-Dindo grading system [14]. The surgical conversion included: 1) RPN converted to conventional laparoscopic partial nephrectomy or open surgery; 2) RPN or OPN converted to radical nephrectomy.

### Study quality assessment

The level of evidence was rated for each included study according to the most updated criteria provided by the Center for Evidence-Based Medicine in Oxford, UK [15]. The methodological quality of RCTs was scored with the Jadad composite scale, which is a 5-point scale [16], [17]. A score of 2 or less indicates low-quality while 3 or more high-quality [16], [18]. The methodological quality of non-RCTs was assessed with the modified Newcastle-Ottawa Scale (NOS) [19], [20], which is a “star system” and ranges between zero up to nine stars.

### Statistical analysis

A meta-analysis was performed to assess the outcomes of RPN when compared with OPN. Odds ratio (OR) or Risk ratio (RR) and mean difference (MD) or standardized mean difference (SMD) was used for binary variables and continuous parameters, respectively. For studies presenting continuous data as medians and range values, means and standard deviations (SD) were calculated using the methodology described by Hozo et al [21] in keeping with the Cochrane Handbook [22]. Pooled estimates were calculated with fixed-effect model (Mantel-Haenszel method) [23] if no significant heterogeneity was detected, otherwise, random-effect model (DerSimonian-Laird method) [24] was used. The pooled effects were determined by Z-test and αof 0.05 was used for statistical significance. The Cochrane *X* ^2^-test and Inconsistency (I^2^) [25] were used to evaluate the heterogeneity among studies. *P*<0.10 indicated the presence of heterogeneity, I^2^<50% indicated acceptable heterogeneity. Publication bias was assessed by a funnel plot. Review Manager software (RevMan 5.1, Cochrane Collaboration, Oxford, UK) was used for analysis.

## Results

### Characteristics and methodological quality of included studies

**Figure 1:**
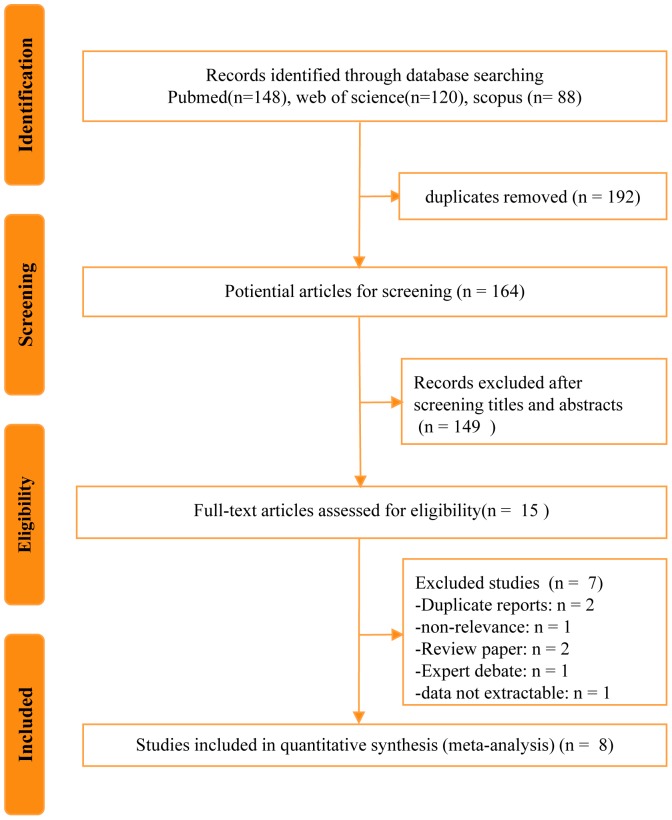
Flowchart for records selection process of the meta-analysis.

The literature search yielded 164 studies, of which 8 were selected in the final analysis including 3418 cases (757 cases for RPN and 2661 cases for OPN) (Fig.1). No additional records were identified through the manual searches of the references cited for these included studies. Table 1 presents the baseline characteristics of the studies. Four studies compared the outcomes of OPN, LPN and RPN [26]-[29]. The majority of studies reported on their single center's experience with RPN compared with OPN, Minervini et al [12] conducted a domestic multi-institutional analysis and Yu et al [29] assessed the NIS database and assessed the use, costs and comparative effectiveness of robotic assisted, laparoscopic and open urological surgery with a propensity adjusted comparison of RPN versus OPN. The patient recruitment periods were mostly between 2005 and 2010. The continuous parameters in the study by Simhan et al had to be pooled as two separate groups because the comparisons in the original study were stratified by moderately and highly complex tumor nephrometry scores [13].

**Table 1:** Characteristics of included studies and quality assessment.

Study	Level of evidence	Design	NO. of centers	Recruitment period	NO. of surgeons	Matching*	Follow-up#, mo RPN/OPN	Quality score§
Alemozaffar et al 2013 [26]	3	R	Single	2008.11–2010.12	Multiple	2,3,4,5,6,7	Perioperative	5 of 9
Laydner et al 2013 [27]	3	R	Single	2009.1–2010.12	Four	1,3,5,8	Perioperative	6 of 9
Lee et al 2011 [10]	4	R	Single	2003.5–2010.12	Multiple	1,2,5,6,8	NA	6 of 9
Lucas et al 2012 [28]	3	R	Single	2004–2010	Single	1,2,3,6,7,8,9	9.4/21.1	6 of 9
Masson-Lecomte et al 2013 [11]	3	RP	Single	2008–2010	Two	1,2,3,4,6,8	19/32	7 of 9
Minervini et al 2013 [12]	2	PN	Domestic multicenter	2010.1–2011.12	Multiple	1,2,3,4,7,8	Perioperative	7 of 9
Simhan et al 2012 [13]	3	RP	Single	2007–2010	Multiple	1,2,3,4,7	17.6/22.7	6 of 9
Yu et al 2012 [29]	2	RP	NIS, >1000 centers	2008.10–2008.12	Multiple	Propensity adjusted	NA	8 of 9

R = retrospective; RP = retrospective analysis, prospective data collecting; PN = prospective non-randomized design; NIS = USA national inpatient sample; NA = not available.

*Matching: 1 = age; 2 = gender; 3 = body mass index; 4 = American Society of Anesthesiologists score; 5 = tumor laterality; 6 = tumor size; 7 = nephrometry score (RENAL or PADUA); 8 = pre-op eGFR; 9 = single surgeon.

# Mean or median.

§ using modified Newcastle-Uttawa Scale (NOS) [19], [20].

Among the included studies, there was one prospective non-randomized comparative study (level of evidence: 2) [12]; 6 retrospective studies compared contemporary of patients (level of evidence: 3) [11], [13], [26]–[28]with the level of 1 study graded up for its large effect size(level of evidence: 2) [29]; and 1 retrospective study used a historical series as the control (level of evidence: 4). 3 retrospective studies declared prospective data collection [11], [13], [29]. The methodological quality of included studies was relatively high. None of the studies were randomized or blinded, with allocation usually at the discretion of the surgeon. Matching criteria between RPN and OPN groups were variable and only 3 studies [11], [13], [28]provided the duration of follow-up for both groups.

### Patient demographics

Table 2 depicts the demographics of the included studies including number of patients, patient age, gender, body mass index (BMI), laterality, tumor size, and malignant/benign pathology ratio. There was no significant difference between the two groups for any of the demographic parameters except for the gender (OR: 0.78, 95% CI 0.62–0.99; p = 0.04), and the tumor size, which was larger for the OPN group (weighted mean difference [WMD]:−0.74; 95% confidence interval [CI], −1.11 to −0.37; p<0.0001). Six studies reported on the kidney tumor nephrometry score (RENAL or PADUA) as a continuous or ordinal outcome [11]–[13], [26]–[28].

**Table 2:** Patient demographics of robotic versus open partial nephrectomy.

Study	Patients, no,RPN/OPN	Age, yr, RPN/OPN	Male:Female,RPN/OPN	BMI, kg/m^2^,RPN/OPN	Right:Left,RPN/OPN	Tumor size, cm RPN/OPN	Malignant: benign^$^ RPN/OPN
Alemozaffar et al 2013 [26]	25∶25	55.9±11.7/61.9±10.1	15∶10/19∶6	27.5±3.8/30.1±5.9	12∶13/17∶8	2.5±1.0/3.3±1.4	NA
Laydner et al 2013 [27]	145∶133	58.5±16.2/55.8±19.7	83∶62/96∶37	35±12.9/30.3±8.2	72∶73/72∶61	3.6±2.4/5.4±3.7	NA
Lee et al 2011 [10]	69∶234	53.5±11.9/54.4±12.8	50∶19/164∶70	25.5±3.2/24.5±2.8	28∶41/107∶127	2.4±1.3/2.6±1.4	64∶5/215∶19
Lucas et al 2012 [28]	27∶54	62.1/57.6*	19∶8/38∶16	31.4/29.6*	NA	2.4/2.3*	17∶10/44∶10
Masson-Lecomte et al 2013 [11]	42∶58	61.7±10.9/60.8±11.2	22∶20/40∶18	26.9±4.2/26.5±5.6	NA	2.8±1.4/3.1±1.2	34∶8/52∶6
Minervini et al 2013 [12]	105∶198	62.3±11.6/63.8±12.4	69∶36/123∶75	25.7/26.2*	NA	2.8±1.5/3.5±1.8	91∶14/156∶42
Simhan et al 2012 [13]							
Moderate nephrometry group	81∶136	56.6±13.1/58.7±11.2	43∶38/89∶47	30.7±6.7/30.0±7.0	NA	3.0±1.6/3.9±2.0	65∶16/120∶16
High nephrometry group	10∶54	56.1±10.7/59.4±10.8	6∶4/28∶26	30.7±3.5/30.9±6.8	NA	3.8±2.3/4.9±3.1	8∶2/49∶5
Yu et al 2012 [29]	253∶1769	Control for factors confounding group assignment with propensity score adjustment					

*median.

### Meta-analysis results

#### 1. Complications, transfusions and conversions

**Figure 2:**
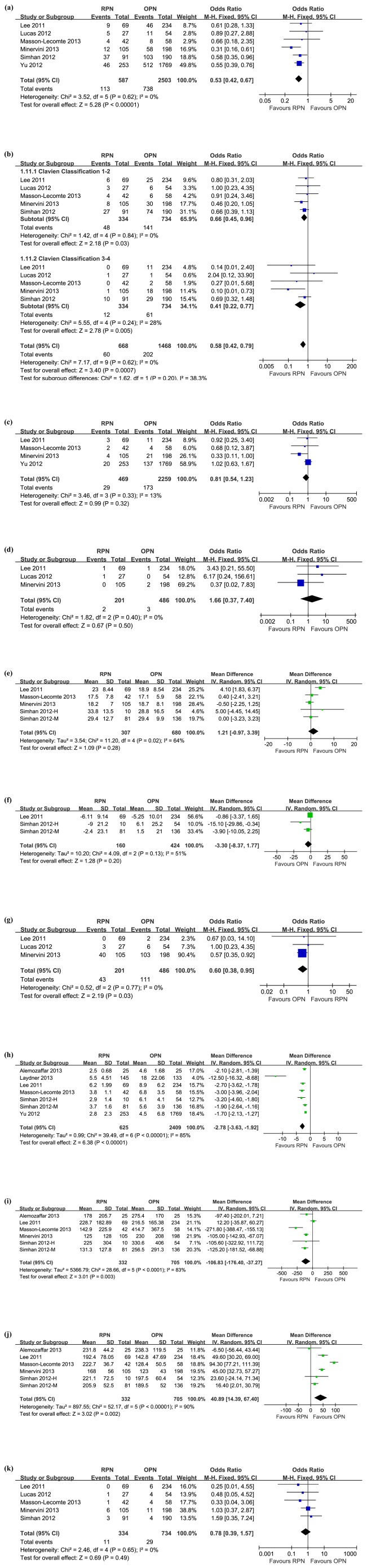
Forest plots of surgical outcomes. (a) overall complications; (b) postoperative complications divided into Clavien grade 1–2 and 3–4; (c) transfusions; (d) conversions to radical nephrectomy; (e) ischemia time; (f) estimated GFR change; (g) unclamping rate; (h) length of stay; (i) estimated blood loss; (j) operative time; (k) positive margins. The following studies are cited: Alemozaffar et al 2013 [26], Laydner et al 2013 [27], Lee et al 2011 [10], Lucas et al 2012 [28], Masson-Lecomte et al 2013 [11], Minervini et al 2013 [12], Simhan et al 2012 [13], Yu et al 2012 [29].

The overall complication rate was significantly lower for the RPN group by pooling the data from 6 studies [10]–[13], [28], [29] that investigate perioperative complications in 3090 patients (19.3% for RPN and 29.5% for OPN; OR: 0.53; 95%CI, 0.42–0.67; p<0.00001) (Fig.2a). Intraoperative complication rate was available for 3 studies [10], [12], [28], and no significant differences were detected between the two groups (3.5% for RPN and 4.9% for OPN; OR: 0.69; 95%CI, 0.29–1.62; p = 0.39). 5 studies [10]–[13], [28] including 1068 patients evaluated postoperative complication rates and all further divided into minor (Clavien Classification 1–2) and major (Clavien Classification 3–5) complications. The pooling data favored the RPN groups for overall postoperative complications (OR: 0.51; 95%CI, 0.36–0.72; p = 0.0001), minor complications (OR: 0.66; 95%CI, 0.45–0.96; p = 0.03) and major complications (OR: 0.58; 95%CI, 0.42–0.79; p = 0.0007) (Fig.2b).

There was no significant difference between the two groups in terms of perioperative transfusion rate (OR: 0.81; 95%CI, 0.54–1.23; p = 0.32) (Fig.2c). Four studies [10], [12], [13], [28] reported the conversion rate of RPN to OPN or LPN, and the weighted rate was 1.4% (4/292). Regarding the rates of conversion to radical nephrectomy, the pooled data of 3 studies [10], [12], [28] including 201 RPN patients and 486 OPN patients showed no significant difference between the two groups (0.7% in the RPN and 0.6% in the OPN; OR: 1.66; 95%CI, 0.37–7.40; p = 0.50) (Fig.2d).

#### 2. Ischemia time and eGFR change

There was no statistical difference found between RPN and OPN regarding ischemia time (WMD: 1.21; 95%CI, −0.97 to 3.39; p = 0.20) (Fig.2e) and eGFR change (WMD: −3.30; 95%CI, −8.37 to 1.77; p = 0.20) (Fig.2f). 3 studies [10], [12], [28] reported the intraoperative renal artery unclamping rate and the pooled data revealed a significantly higher rate in the OPN group than the RPN group (OR: 0.60; 95%CI, 0.38–0.95; p = 0.03) (Fig.2g).

#### 3. Length of hospital stay, estimated blood loss and operative time

There was a significantly shorter postoperative hospital stay in the RPN group (WMD: −2.78; 95%CI, −3.36 to −1.92; p<0.00001) (Fig.2h) and less estimated blood loss in this same group (WMD: −106.83; 95%CI, −176.4 to −37.27; p = 0.003) (Fig.2i), whereas the operative time was statistically longer in the RPN than OPN (WMD: 40.89; 95%CI, 14.39–67.40; p = 0.002) (Fig.2j).

#### 4. Margin status, tumor recurrence and metastasis

Five studies [10]–[13], [28]reported the margin status of surgical specimens. There was no significant difference regarding positive margin rates between the two groups by pooling the data of the 1068 patients in these five studies (OR: 0.78; 95%CI, 0.39–1.57; p = 0.49) (Fig.2k). Although four studies [10], [11], [13], [28] reported the tumor recurrence or metastasis rate and a trend was observed toward a higher failure of cancer control rate for OPN (2.2% versus 0.4%), the data were not pooled for meta-analysis for the differences in the length of follow-up duration between the studies.

#### 5. Cost analysis

Three studies compared costs associated with partial nephrectomy using robotic and open approaches, pooling the data of 2350 patients in these three studies showed no statistical difference between the RPN and OPN groups (WMD: −3115; 95%CI, −8053 to 1822; p = 0.22).

#### 6. Reporting bias analysis

**Figure 3:**
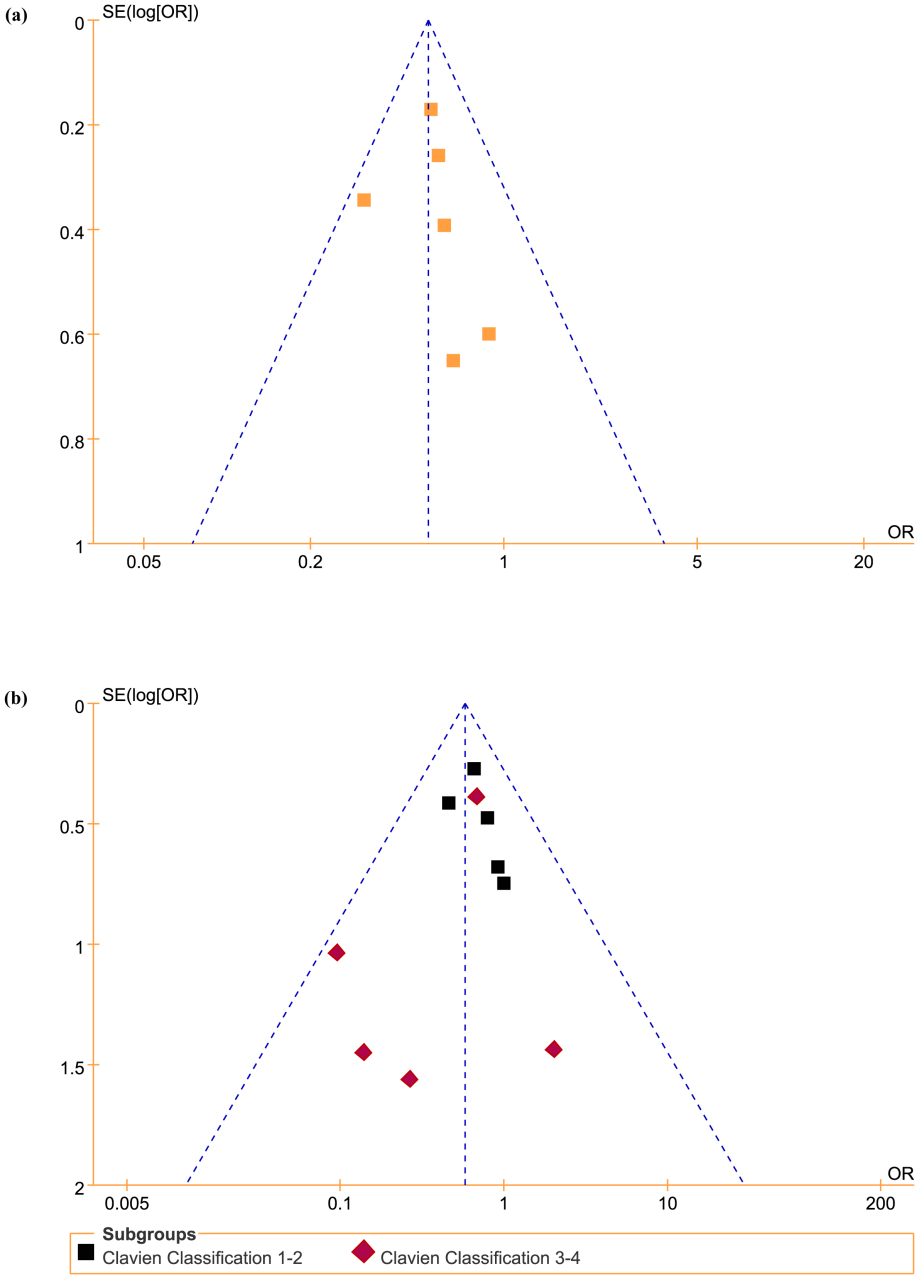
Reporting bias analysis. Funnel plots of the studies included in this meta-analysis reporting overall complication rates (a) and postoperative complications with Clavien grade classifications (b). SE = standard error; OR = odds ratio.

Figure 3 shows funnel plots of the studies included in this meta-analysis reporting perioperative complication rates. All studies lie inside the 95% confidence intervals, with an even distribution around the vertical, indicating no obvious reporting bias.

## Discussion

The present meta-analysis pooled the current available evidences comparing RPN with OPN in the treatment of the localized kidney tumor. No significant difference was observed between the two techniques regarding baseline demographics, except for gender and the tumor size, the weighted mean of which was lower for tumors treated with RPN. This can be regarded as a selection bias, and explained partially by the fact that the system was initially recommended for small masses. In the study of a two-year observational multicenter outcomes research of RPN versus OPN by Minervini et al, the clinical tumor size was significantly different between the two groups, whereas the tumor nephrometry score measured by PADUA was comparable [12]. Nephrometry scoring allows for systematic analysis of anatomical renal tumor characteristics and has an important role in PN outcomes reporting because it indicates the degree of technical complexity and allows for valid comparison among different cohorts. As to the surgical outcomes, no significant difference was observed regarding the transfusion rate, surgical conversion rate, ischemia time, and positive margin status.

The incidence of perioperative complications was significantly lower following RPN(19.3% versus 29.5%; p<0.00001). In the subgroup analysis of postoperative complications, both minor and major complications more frequently occurred following OPN(14.4% versus 19.2%, p = 0.03 and 3.6% versus 8.3%, p = 0.0007, respectively), whereas there was no Clavien grade 5 (death) complication in either group. Lucas et al reported one grade 4a complication in the RPN group (stroke) [28]. In the largest single center non-comparative series to date, which consists of 400 patients undergoing RPN, there were 61 cases (15.3%) of postoperative complications, which were mainly low grade (Clavien grade 3-4 in 3.2%) [30]. In the open group, Simhan et al reported that major complications requiring a secondary procedure developed in 29 patients (15.3%) [13]. In the multi-institutional comparative study, Minervini et al demonstrated that open surgical approach is the only independent risk factor associated with Clavien grade 3–4 complications [12].

The other encouraging finding in favor of robotic surgery was the shorter length of hospital stay, with a difference reaching statistical significance between the two groups. Besides, we unexpectedly found that the cost of RPN was not significantly higher than that of OPN (p = 0.22). Laydner et al assessed the Cleveland Clinic data and found that the increased cost of RPN due to instrumentation and supplies can be offset by decreased cost of hospitalization compared with the OPN group [27]. Yu et al used a population based approach to compare the perioperative costs of robotic, laparoscopic, and open surgery in urology. They found that health costs were higher for robotic vs laparoscopic and open surgery for all procedures except for partial nephrectomy, where costs were similar [29]. In this regard, post-discharge convalescence and return to work analysis should be included in the future study, which may have some impacts on the social cost.

The pooled data of operative time showed a significant difference between the two techniques, which was longer for the RPN (WMD: 40.89; 95%CI, 14.39–67.40; p = 0.002). The observed difference of approximately 40 min can be pertaining to the preparation and docking of the robot. Masson-Lecomte et al found the difference become insignificant when “skin-to-skin” time compared (excluding the setup and docking time), whereas the total operating room occupation was more than 100 min over the operative time itself for RPN [11]. Furthermore, many studies found that the surgeon experience also could affect the overall operative time.

There was a significant lower amount of estimated blood loss in the RPN group. However, in the present study, we found that there were more unclamping cases in the OPN (p = 0.03). This fact may have biased the parameter of estimated blood loss favoring RPN. The pooled data showed comparable ischemia time, postoperative renal function impairment and positive margin rate between the two techniques, which was also true in patients with moderate or high complex tumors, as reported by Simhan et al [13]. Although a trend was observed toward a higher failure of cancer control rate for OPN (2.2% versus 0.4%), it is not appropriate to estimate the weighted effect with the odds ratio of tumor recurrence and metastasis for the differences in the length of follow-up duration between the studies. A total of 11 cases of tumor recurrence and one of metastasis reported in OPN; while in the RPN group, one patient had metastatic disease within 1 year of surgery and no one patient experienced a local recurrence (including the eleven patients with a positive surgical margin) [10], [11], [13]. Lee et al report two OPN patients with a positive margin status had evidence of recurrent masses treated with radiofrequency ablation [10]. Nonetheless, the follow-up period was limited in the included studies, especially for the RPN patients, so long-term oncologic outcomes of RPN compared to OPN remain to be determined.

All dichotomous outcomes had a low heterogeneity, but which was significant for most of the continuous variables. Factors of different sample sizes, multiple surgeons with different surgical experience, tumor complexity, the variety of ischemia (warm, cold, and no ischemia) may contribute to the study-between-study heterogeneity. The fact that none of the RPN series in the included studies were totally performed within the surgeon's learning curve (first 25 cases as reported by Haseebuddin et al. [31]) might reduce the effect of heterogeneity.

The main limitation of the present meta-analysis is that all the included studies were retrospective non-randomized comparisons, except one prospectively derived comparative study. Ideally, every surgical option for the treatment of the renal masses should be compared to OPN which is the best matching standard and has robust data regarding surgical and oncological outcomes. Although it is laudable to call for prospective randomized studies comparing RPN with OPN, with true clinical equipoise, consisting of a homogeneous group of patients with renal masses, such studies are difficult to carry out in the real world. This review was performed timely and impartially, and conducted systematically and methodologically in accordance with Cochrane standards. This year is the 20^th^ anniversary of LPN [32] and the 10^th^ anniversary of RPN [33], while open surgery currently remains as a standard of care for partial nephrectomy in the 2013 European Urology guidelines on renal cell carcinoma [1]. The underlying reason is that RPN effects on long-term renal preservation and cancer-control compared with OPN are to be defined. Well-designed global multi-center studies with extensive follow-up are awaited before a new standard surgical approach of PN can be established.

## Conclusions

Meta-analysis of the currently available evidence comparing RPN and OPN reveals that robotic technique results in a significantly lower rate of perioperative complications, less estimated blood loss with shorter hospital stay, albeit with a longer operative time, while transfusions, ischemia time and estimated GFR change, early cancer outcomes, and overall cost are similar to the open surgery. Given the inherent limitations of the included studies, however, well-designed prospective randomized controlled trials are needed to confirm and update our findings.

## Supporting Information

Checklist S1 PRISMA 2009 Checklist.
(DOC)
